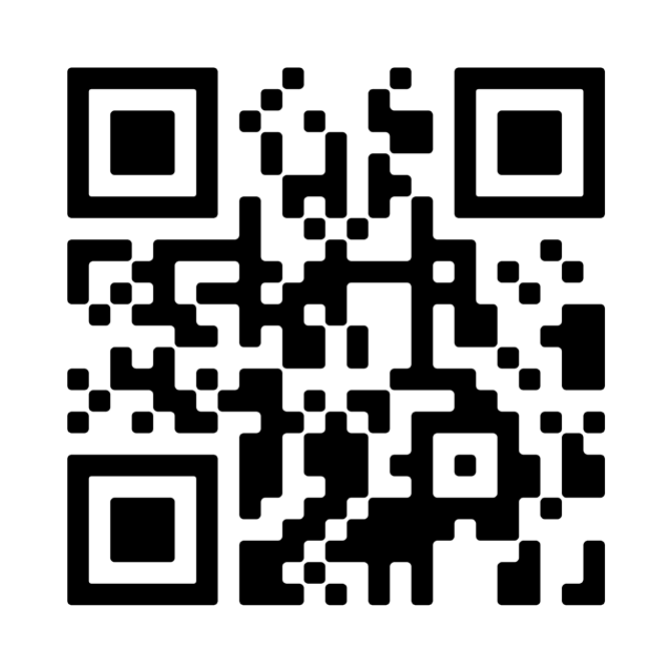# Implementation of robot-assisted myomectomy in a large university hospital: a retrospective descriptive study

**DOI:** 10.52054/FVVO.15.3.089

**Published:** 2023-09-24

**Authors:** M Tahapary, S Timmerman, A Ledger, K Dewilde, W Froyman

**Affiliations:** Department of Obstetrics and Gynecology, University Hospital Leuven, Leuven, Belgium; Afdeling Gynaecologie, Medisch Spectrum Twente, Enschede, The Netherlands; Department of Development and Regeneration, KU Leuven, Leuven, Belgium

**Keywords:** Myoma, leiomyoma, fibroid, myomectomy, robot assisted surgery, robot-assisted myomectomy

## Abstract

**Background:**

Myomectomy is often the preferred treatment for symptomatic patients with myomas who wish to preserve their fertility, with a shift from open surgery towards minimally invasive techniques.

**Objectives:**

Retrospective study assessing patient and surgery characteristics, follow-up, and outcomes of robot-assisted myomectomy (RAM) and abdominal myomectomy (AM) in women treated between January 1, 2018, and February 28, 2022, in a Belgian tertiary care hospital.

**Materials and Methods:**

A descriptive analysis was conducted on consecutive patients who underwent myomectomies. 2018 was considered the learning curve for RAM.

**Main Outcome Measures:**

We assessed rate of open surgery, operation time, postoperative hospital stay, and operative complications.

**Results:**

In total, 94 RAMs and 15 AMs were performed. The rate of AMs was 56.5% in 2018 versus 2.3% after the learning curve. The median operation time for RAM was 136.5 minutes and 131 minutes for AM. Conversion rate for RAM was 0%. The median postoperative hospital stay after RAM was 1 night and 4 nights for AM. Postoperative complication rate was low, with only 14.9% and 33.3% of patients requiring pharmacological treatment of complications after RAM or AM, respectively. No surgical re-intervention was needed in any group.

**Conclusions:**

Implementation of RAM at our centre resulted in a significant reduction of open surgery rate. RAM demonstrated shorter hospital stays and a lower incidence of complications compared to AM.

**What is new?:**

Our study highlights the successful adoption of RAM, showcasing its potential to replace AM even in complex cases. The findings affirm the safety and feasibility of RAM, supporting its use as a valuable technique for minimally invasive myomectomy.

## Introduction

Uterine myomas, also known as fibroids or leiomyomas, are benign myometrial tumours that represent the most common tumours of the female reproductive tract. Prior to menopause, approximately 70-80% of women are likely to develop uterine myomas, of whom 25% to 50% will present with clinical symptoms (Williams et al., 2006). Myomas vary in size, number, and location based on the FIGO classification (Munro et al., 2011). Uterine myomas may not only induce (non) cyclic pain and abnormal uterine bleeding (heavy, irregular and prolonged bleeding) but also bladder or bowel dysfunction, dyspareunia, bulk symptoms, impaired fertility, pregnancy complications, and adverse obstetric outcomes (Pritts and Olive, 2012; Stewart et al., 2017). Hence, myomas in symptomatic women may significantly impact the physical, social, emotional, and material quality of life (Downes et al., 2010; Fortin et al., 2018). A wide array of treatment options exists, ranging from medical therapy to various surgical interventions (Allaire et al., 2015; Donnez and Dolmans, 2016; Lukes et al., 2019; Pérez-López et al., 2014). Medical or expectant management can be an option for asymptomatic women, women with mild symptoms, or women close to menopause (Laughlin and Stewart, 2011). Medical treatment, either hormonal or non- hormonal, primarily aims at symptom control rather than eradicating myomas. Other therapeutic options may include uterine artery embolization (UAE), high-intensity focused ultrasound (HIFU) ablation, radiofrequency ablation (RFA), myomectomy or hysterectomy (Stewart, 2015). Hysterectomy remains the definitive treatment for myomas, but this treatment is not an option for women who wish to preserve their fertility. Due to the young age of many patients with myomas, fertility is often a concern, creating a higher demand on uterine sparing options. In case medical or expectant treatment fails, myomectomy will be the treatment of choice for well-defined lesions as myomas in patients who still want children. The preferred surgical approach for myomectomy can be based on patient characteristics (e.g., BMI, previous abdominal surgery) and myoma characteristics (e.g., number, size, FIGO classification). Laparotomic myomectomy generally has shorter operation times, provides better accessibility for multiple or posterior myomas, but leads to longer hospitalisation time and more postoperative adhesions compared to minimally invasive techniques. Laparoscopy minimizes intraoperative blood loss, postoperative pain, length of hospital stay and scar burden compared to laparotomy, but might provide less accessibility and more difficulties in adequately suturing uterine incisions (Lavazzo et al., 2016; Vargas et al., 2019; Putra et al., 2021). Robot-assisted surgery (RAS) is a relatively new surgical innovation, revolutionising minimally invasive surgery. RAS enables the surgeon to conduct the operation from a computer console, situated away from the surgical table.

RAS provides a variety of technical advantages such as joint-wristed instruments, motion scaling, tremor elimination, a stable 3-dimensional vision, greater precision in dissection, easier suturing and knot tying, shorter learning curve, and favourable surgical ergonomics (Aendekerk et al., 2019; Herrinton et al., 2020; Liu et al., 2014; Winter et al., 2015).

In 2019, Aendekerk et al. (2019) reported on the first series of RAM performed in our centre. They showed a decrease in open surgery and laparoscopy in favour of RAM. However, at the time of this previous study, only one robotic platform was available for the whole hospital, resulting in restricted robotic surgery time.

The purpose of this retrospective analysis is to describe the patient and surgery characteristics, and short-term postoperative outcomes of patients who underwent robot-assisted myomectomy (RAM) or abdominal myomectomy (AM). The study involves the adoption of RAM by a single surgeon new in robotic surgery. Thereby, we will assess the learning curve for RAM and the effect of implementing RAM on the rates of open surgery in our centre.

## Patients and methods

We performed a single centre retrospective analysis including consecutive patients who underwent myomectomy for symptomatic myomas in the period between January 1st 2018 and February 28th, 2022, in a tertiary care hospital. All patients underwent a transvaginal ultrasound evaluation preoperatively. Myomectomies were performed either through AM or RAM, the latter were performed by the same surgeon (WF). Since 2018 two robotic platforms are available in our hospital, allowing us more robotic access for benign gynaecology. No conventional laparoscopic myomectomies were performed during the study period in order to optimise the RAM learning curve. The first year after the implementation of RAM was considered to be the learning curve (i.e., 2018).

**Figure 1:**
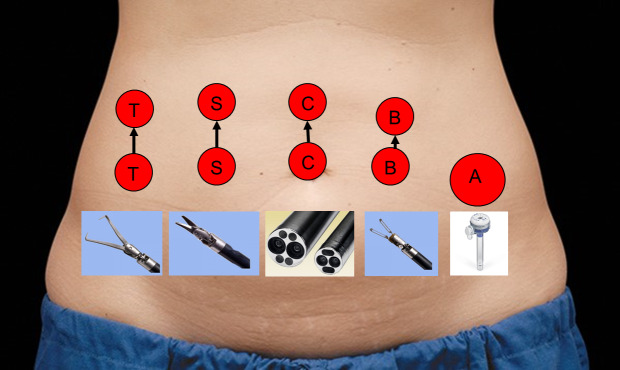
Port placement. T: tenaculum; S: monopolar scissors; C: camera; B: bipolar grasper; A: assistant port.

The Da Vinci robotic surgical system model type Xi (Intuitive Surgical Inc. Sunnyvale, CA) was used to perform RAMs. The surgical technique for RAM in our centre can be observed in video 1, with minimal adaptations compared to the technique of Arian et al (Arian et al., 2017). Patients should be counselled that when the uterus is very large, port placement often must be adapted, with ports placed higher in the abdominal wall as to not limit manoeuvring space (Figure 1).

Haemostatic measures were not routinely performed but included local infiltration with vasopressin-analogues or systemic administration of tranexamic acid in some cases.

For tissue extraction, in-bag scalpel morcellation, with paper-roll technique (Moawad et al., 2019), was the preferred method, making power morcellation obsolete.

Data included patient characteristics (age, BMI, menopausal state, symptoms), myoma characteristics (type, location, dimension of largest myoma), surgical information (operation time, blood loss, weight of resected tissue), and postoperative follow-up (hospitalisation length, need for postoperative blood transfusion and complications up to 6 weeks postoperatively according to the Clavien-Dindo classification system (Clavien et al., 2009)). Surgical data were all prospectively registered in the operation report. Data was collected by review of the medical records and a descriptive analysis was performed. For this analysis, we stratified patient and myoma characteristics by type of surgery. Surgical information and postoperative follow-up were stratified by type of surgery and also per year. The study protocol (S64834) was approved by the Ethics Committee Research UZ / KU Leuven.

## Results

In total, 94 RAMs and 15 AMs were performed. Indications for myomectomy included menorrhagia, dysmenorrhoea, bulk symptoms, or infertility. Patient characteristics are described in Table I and include the age at time of surgery, menopausal state, and BMI. No difference in BMI was observed between the two groups. Note however that the maximum value for BMI in the RAM group was 42.6 versus 32.0 in the AM group.

**Table I:** Patient characteristics.

	RAM (n=94)	AM (n=15)
**Patient age at time of surgery**		
Median	35.50	36
Range	26-55	23-46
**Menopausal state**		
Premenopausal	93 (99%)	15 (100%)
Postmenopausal	1 (1%)	0
**BMI**		
Median	23.25	23.53
Range	16.5-42.6	17.3-32
Missing	1 (1%)	0
**Symptoms**		
Abnormal uterine bleeding	39 (41.5%)	4 (26.7%)
Bulk symptoms	48 (51.1%)	8 (53.3%)
Fertility issue	35 (37.2%)	4 (26.7%)
**History**		
Previous pelvic surgery	28 (29.7%)	1 (6.6%)
Previous myomectomy	9 (9.6%)	0
History of endometriosis	8 (8.5%)	0 (0%)
History of PID	3 (3%)	2 (13%)
**Increased bleeding tendency**	4 (4.2%)	1 (6.7%)
**Previous use of SPRMs**	8 (8.5%)	1 (6.7%)

BMI: body mass index; PID: pelvic inflammatory disease; SPRMs: selective progesterone receptor modulators.

In the RAM and AM group, abnormal uterine bleeding was reported in 41.5% and 26.7%, bulk symptoms in 51.1% and 53.3%; and fertility issues in 37.2% and 26.7% respectively. Previous pelvic surgery was registered in 29.7% and 6.6% in the RAM and AM group, respectively. In the RAM group, 9.6% had undergone previous myomectomy versus 0% in the AM group. Myoma characteristics are described in Table II and include diameter of largest myoma on ultrasound, FIGO classification, and myoma location. In the RAM group the diameter of largest myoma on ultrasound was 159mm versus 160mm in the AM group. Data about myoma characteristics are comparable between the two groups.

**Table II:** Myoma characteristics.

	RAM (n=94)	AM (n=15)
**Number of myomas resected per case**		
1	49	6
2-5	39	7
>5	6	2
**Size of largest myoma per case (mm)**		
Median	73	96
Range	24-159	53-160
**Total number of myomas**	204	44
**Location of the myoma (total)**		
Anterior	44 (22%)	2 (5%)
Posterior	53 (26%)	5 (11%)
Fundal	39 (19%)	1 (2%)
Mix	61 (30%)	36 (82%)
Other	7 (3%)	0
**FIGO class of the myoma (total)**		
1	1 (0.5%)	0
2	20 (10%)	5 (11%)
3	13 (6%)	6 (14%)
4	68 (33%)	15 (34%)
5	24(12%)	2 (5%)
6	56 (28%)	14 (32%)
7	21 (10%)	2 (5%)
8	1 (0.5%)	0

BMI: body mass index; PID: pelvic inflammatory disease; SPRMs: selective progesterone receptor modulators.

An overall view of descriptive statistics, not stratified by year, is presented in Table III. Descriptive statistics of the main outcomes of interest for the RAM group are presented in Table IV.

**Table III:** Overall outcome measures, not stratified by year.

Variable	RAM (n=94)	AM (n=15)
EBL mL (median)	275	500
Operation time in minutes (median)	136.5	131
Complications (CD>1) %	15% (14)	33.3% (5)
Hospital stay in nights (median)	1	4
Weight of resected tissue in gr (median)	163.5	610

EBL: estimated blood loss; CD: Clavien-Dindo.

**Table IV:** Descriptive statistics of the outcomes of interest for the RAM group.

Variable		RAM				
Year		2018 (n=10)	2019 (n=18)	2020 (n=28)	2021+2022 (n=38)	2019, 2020, 2021+2022 combined (n=84)
EBL mL (median)		175	115	350	400	300
Operation time in minutes (median)		176.5	126	136	138.5	134.5
Complications (CD >1) %		1 (11%)	2 (12.5%)	3 (12%)	8 (21%)	13 (15.4%)
	Post-operative hypertension requiring medication.	1 (11%)	1 (5.6%)	0	0	
	Peri-operative blood transfusion	0	1 (5.6%)	3 (12%)	3 (7.9%)	
	Superficial surgical site infection, treated with antibiotics.	0	0	0	3 (7.9%)	
	Abdominal discomfort, treated with empirical antibiotics.	0	0	0	1 (2.6%)	
	Vaginal bleeding 2 weeks post-operatively, treated with tranexaminic acid.	0	0	0	1 (2.6%)	
Hospital stay in nights (median)		3	2	1.5	1	1
Weight of resected tissue in gr (median)		164	59	242	151	163

EBL: estimated blood loss; CD: Clavien-Dindo.

**Figure 2:**
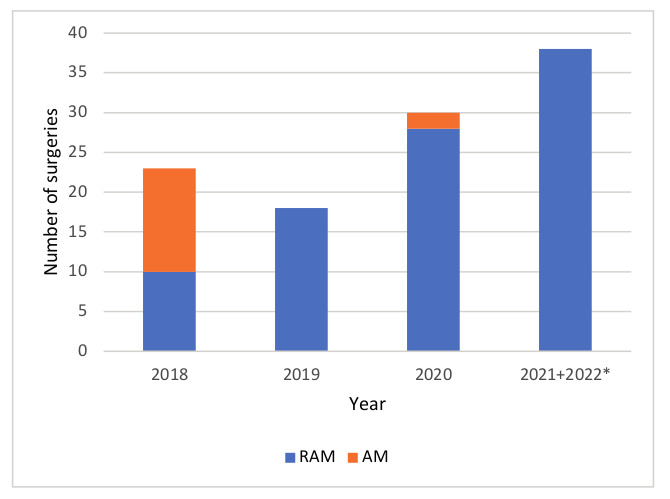
Rate of AM and RAM. 6 patients were included in the beginning of 2022(*).

**Figure 3:**
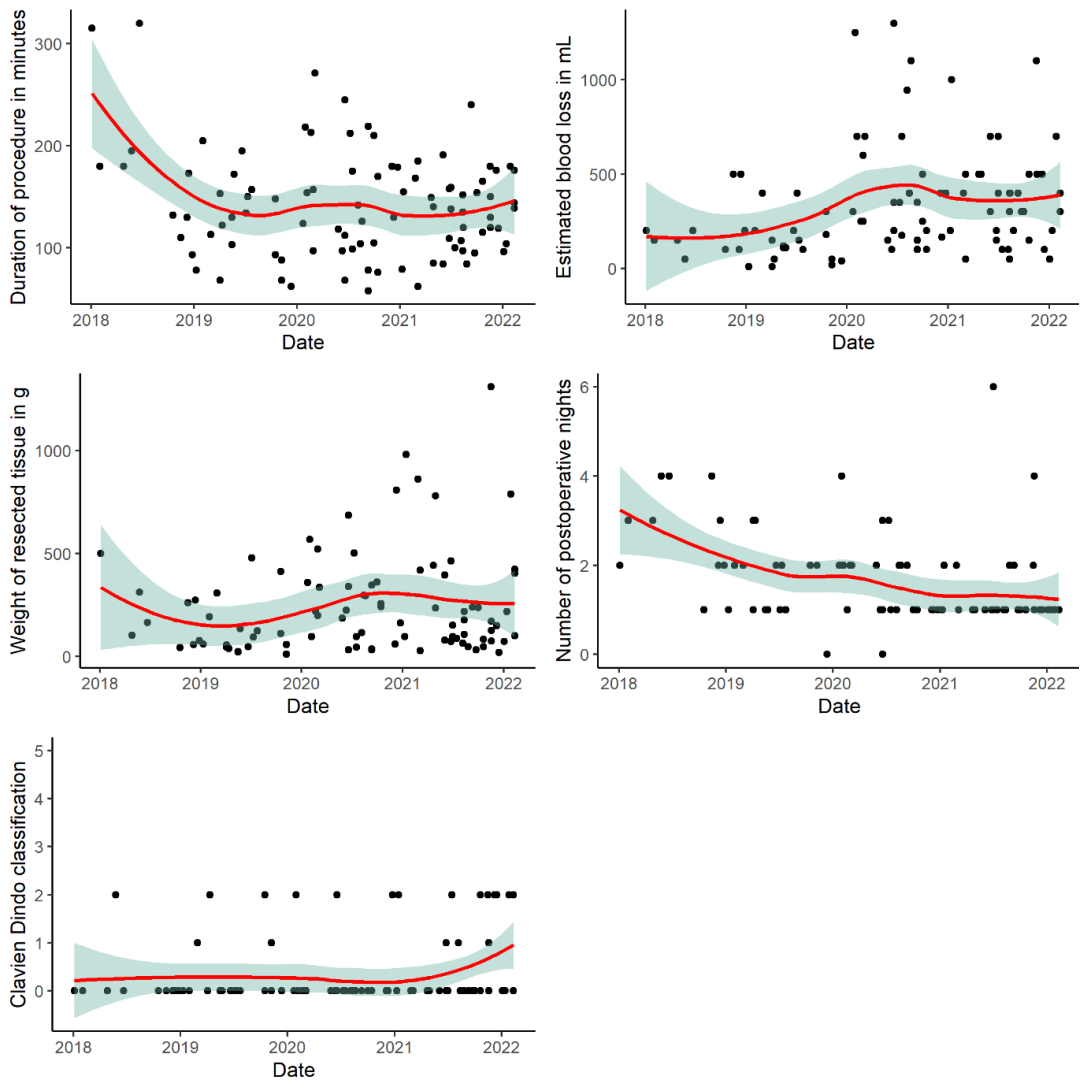
Outcomes of interest for RAM evolved over the years during implementation and learning curve. The scatterplot (black dots) describes individual case outcomes, with a LOESS (locally weighted smoothing) smooth curve to show relationship (red line). The green area is the 95% CI of this LOESS curve.

The rate of AMs in 2018 was 56.5% versus 2.3% after the learning curve as shown in Figure 2. Figure 3 shows plots depicting how the main outcomes of interest for RAM evolved over the years during implementation and relative learning curve.

Within the last 1.5 year only RAMs were performed, without decrease in pre-operative myoma size or the weight of resected tissue (with a maximum of 1320 g for AM and 1308 g for RAM). The median operation time for RAM was 136.5 minutes and 131 minutes for AM. Operation time for RAM shortened from a median of 176.5 minutes in 2018 to a plateau of 136.5 minutes over the following years after the learning curve. The conversion rate for RAM was 0%. Estimated blood loss (median) was 275mL for RAM and 500mL for AM. Opening of the uterine cavity occurred in 28.7% of patients in the RAM group and in 13.3% of patients in the AM group. The median postoperative hospital stay after RAM and AM was 1 and 4 nights, respectively. Postoperatively 33.3% and 14.9% of patients required pharmacological treatment of complications (Clavien-Dindo grade 2) after AM or RAM, respectively. Three patients who underwent AM (20%) and 6 patients who underwent RAM (6.3%) required a blood transfusion after their respective surgeries. Surgical re-intervention was not required in any group.

## Discussion

Our single centre study on the implementation of robot-assisted myomectomy showed a shift towards minimally invasive surgery and a shortening of operation time as the RAM learning curve progressed. Very few complications and no conversions were registered in our cohort, regardless of the myoma size.

To the best of our knowledge, this is the largest European cohort of RAM, investigating the adoption of the technique by a single surgeon new in robotic surgery. A limitation of this study is the retrospective design. However, missing data was minimal, as clinical, ultrasound and surgical data were routinely registered for all patients during the pre-operative anaesthesiology, ultrasound consultation and in the structured operation report. Another limitation is the lack of patients undergoing laparoscopic myomectomy in this cohort, explained by our temporary stop of performing laparoscopic myomectomy in order to provide a sufficient number of patients eligible for RAM during the learning curve (without charging to additional costs of robotic surgery to the patient). While this improved learning curve in RAM allowed us to drastically reduce the open surgery rate, we believe that there remains also an important place for conventional laparoscopy, especially given the costs and limited access to robotic systems. In a previous study performed in our centre, including also cases undergoing laparoscopic myomectomy, Aendekerk et al. (2019) provided an algorithm for clinicians to decide upon the best modus operandi of myomectomy based on various volumes and types of myomas. Laparoscopic myomectomy was suggested for single myomas up to 70 mm or multiple myomas with a cumulative diameter up to 150mm, robot- assisted myomectomy for single myomas of 80 to 110 mm, and laparotomic myomectomy for single myomas of 120 mm or larger and multiple myomas with a cumulative diameter more than 200 mm. After implementing robot-assisted surgery, Aendekerk et al. (2019) reported a decrease in AM from 34.7% to 20.2% and laparoscopic myomectomies from 65.3% to 28.8% after introduction of RAM (rising to 51%). However, during that study period, only one robotic system was available for the whole hospital, resulting in limited access.

Our current study highlights the opportunity to further facilitate offering minimally invasive surgery to patients, with a decrease in AM from 56.5% to 2.3%, after the learning curve, on condition that sufficient robotic access is available. We did not observe a strict upper limit in terms of size to consider RAM, but this is dependent on patient characteristics, such as the morphology of the uterus and pelvis-abdomen, and on the operator. As long as safe placement of the trocars and containment of the specimen in a retrieval bag is possible, RAM will prove to be feasible. The initial number of AM was especially high due to the stop of performing laparoscopic myomectomy in the beginning of 2018.

At the start of the learning curve, only ‘simpler’ cases were selected for robotic myomectomy, afterwards quickly shifting to more complicated cases as shown in the scatterplots (Figure 3), where RAM seems to replace the former AM cases.

Barakat et al. compared surgical outcomes of robot-assisted myomectomy, standard laparoscopic myomectomy, and open myomectomy, and found postoperative bleeding and the need for blood transfusion to be higher in the laparotomy group. In line with the findings of Barakat et al. only 6.4% (6/94) of RAM cases required a blood transfusion, compared to 20% (3/15) in the AM group. In our cohort no cases of severe postoperative bleeding were reported.

A suggested significant disadvantage of RAM was longer operation time (Barakat et al., 2011), although another study did not confirm this when specifically considering experienced surgeons (Herrington et al). As surgical experience with the robot increases, we expect operation time to improve. Our findings showed a shortening of median operation time by 40 minutes following a one-year RAM learning curve, resulting in similar median operation time for AM (131 min) and RAM (136.5 min).

During the study period, the focus on ‘Enhanced Recovery After Surgery’ increased in general in our hospital, both for RAM and AM (Stone et al., 2021). Despite this, the hospital stay for patients undergoing RAM or AM differed markedly in our cohort. Rapid mobilisation and early discharge might have been more stimulated for patients who underwent minimally invasive surgery, resulting in a greater effect in the RAM group.

It is important to remind that myomectomy and especially morcellation should not be advised in cases where malignancy is suspected. In our centre, we use transvaginal ultrasonography as a triage instrument to identify patients who preferably should not undergo morcellation. Some ultrasound features are indicative of a sarcoma while others are for myomas (Amant et al., 2015). In cases of doubt an additional MRI could be considered (Camponovo et al., 2023).

This analysis does not report on the topic of morcellation, however, it should be noted that even enucleation of myomas causes spilling (Sandberg et al., 2016). Takeda et al. reported dispersion of leiomyoma cells even when careful in-bag morcellation was performed (Takeda et al., 2018). However, we believe that by performing in-bag morcellation we can avoid the spread of macroscopic tissue fragments, limiting the risk of patients developing diffuse peritoneal leiomyomatosis (Siedhoff and Cohen, 2017).

## Conclusion

After the introduction of robot-assisted myomectomy in our centre, we noted a marked increase of cases that could be managed in a minimally invasive way with a steep learning curve. After the learning curve, RAM appears to replace almost all AM, with fewer adverse events and shorter hospital stay in our cohort of 109 cases. Future research should involve multicentre prospective studies, focusing on patient reported outcomes, subsequent fertility and pregnancy, and cost-effectiveness of robot-assisted myomectomy in comparison to other approaches.

## Video scan (read QR)

https://vimeo.com/866722286/bdafcb0b5e?share=copy

**Figure:**